# Phenotypic screening for quinolone resistance in *Escherichia coli*

**DOI:** 10.1007/s10096-019-03608-w

**Published:** 2019-06-18

**Authors:** Linus Dellgren, Carina Claesson, Marie Högdahl, Jon Forsberg, Håkan Hanberger, Lennart E. Nilsson, Anita Hällgren

**Affiliations:** 1 Department of Infectious Diseases, Linköping University, Linköping, Sweden; 2 Department of Clinical and Experimental Medicine, Linköping University, Linköping, Sweden; 3 Department of Clinical Microbiology, Linköping University, Linköping, Sweden; 4 Department of Urology, Linköping University, Linköping, Sweden

**Keywords:** PMQR, Susceptibility testing, *E. coli*, Transrectal ultrasound (TRUS)-guided biopsy

## Abstract

Recent studies show that rectal colonization with low-level ciprofloxacin-resistant *Escherichia coli* (ciprofloxacin minimal inhibitory concentration (MIC) above the epidemiological cutoff point, but below the clinical breakpoint for resistance), i.e., in the range > 0.06–0.5 mg/L is an independent risk factor for febrile urinary tract infection after transrectal ultrasound-guided biopsy (TRUS-B) of the prostate, adding to the other risk posed by established ciprofloxacin resistance in *E. coli* (MIC > 0.5 mg/L) as currently defined. We aimed to identify the quinolone that by disk diffusion best discriminates phenotypic wild-type isolates (ciprofloxacin MIC ≤ 0.06 mg/L) of *E. coli* from isolates with acquired resistance, and to determine the resistance genotype of each isolate. The susceptibility of 108 *E. coli* isolates was evaluated by ciprofloxacin, levofloxacin, moxifloxacin, nalidixic acid, and pefloxacin disk diffusion and correlated to ciprofloxacin MIC (broth microdilution) using EUCAST methodology. Genotypic resistance was identified by PCR and DNA sequencing. The specificity was 100% for all quinolone disks. Sensitivity varied substantially, as follows: ciprofloxacin 59%, levofloxacin 46%, moxifloxacin 59%, nalidixic acid 97%, and pefloxacin 97%. We suggest that in situations where low-level quinolone resistance might be of importance, such as when screening for quinolone resistance in fecal samples pre-TRUS-B, a pefloxacin (S ≥ 24 mm) or nalidixic acid (S ≥ 19 mm) disk, or a combination of the two, should be used. In a setting where plasmid-mediated resistance is prevalent, pefloxacin might perform better than nalidixic acid.

**Electronic supplementary material:**

The online version of this article (10.1007/s10096-019-03608-w) contains supplementary material, which is available to authorized users.

## Introduction

Transrectal ultrasound (TRUS)-guided biopsy is a common procedure in urology to examine suspected malignancies of the prostate. The frequency of post-TRUS febrile urinary tract infection (UTI) is 1–6%, varying with population and definition [1–3]. Twenty years ago, it was shown that a single dose of 750 mg ciprofloxacin lowers the frequency of post-TRUS biopsy infections, and this regimen is currently standard prophylaxis in Sweden [1, 4].

Despite this use of a quinolone prophylaxis, in the past two decades, there has been an alarming increase in post-TRUS-B UTIs [5–8]. In studies between 1996 and 2009, the incidence of severe infections increased from 0.5–1 to 2–6% [5, 6]. There is strong evidence suggesting that decreased susceptibility to quinolones in the most common pathogen, *Escherichia coli* (75–90%) is the cause of this increase [3, 5, 8–10].

In addition to patient-related factors, fecal carriage of quinolone-resistant *E. coli* is a risk factor for post-TRUS-B infection [11, 12]. Culturing of *E. coli* from feces and susceptibility testing, with subsequent modification of antibiotic prophylaxis, has been shown to decrease the frequency of post-TRUS-B infection [11]. A recent study by Lee et al. also suggests that such directed prophylaxis may be cost-effective [13].

The European breakpoint committee (EUCAST) designates the clinical breakpoints for different species and antibiotic combinations, defining isolates as susceptible (S), susceptible, increased exposure (I), and resistant (R), using the disk diffusion method and determination of minimal inhibitory concentration (MIC). The clinical breakpoints are based on the expected clinical effect from the recommended dosage (with regard to the site of infection) of an antibiotic relating to bacterial isolates’ susceptibility, expressed as S, I, or R. In addition to clinical breakpoints, EUCAST also determines the epidemiological cutoff point (ECOFF) which is the highest anticipated MIC that a wild-type population, i.e., the population devoid of any phenotypically detectable acquired resistance mechanisms, of a species is expected to have [14].

Screening for quinolone resistance in *Enterobacteriaceae* with nalidixic acid was standard praxis in Sweden until 2010, when it was replaced by screening with ciprofloxacin in accordance with EUCAST recommendations. The breakpoints for ciprofloxacin S and R in *Enterobacteriale*s are MIC ≤ 0.25 mg/L and > 0.5 mg/L, respectively [14]. *E. coli* isolates with ciprofloxacin MIC 0.5 mg/L are categorized as in an area of technical uncertainty (ATU). The ATUs are warnings to laboratory staff that there is an uncertainty that needs to be addressed before reporting AST results to clinical colleagues [14].

However, preliminary data from the Department of Urology, Östergötland County, Sweden, has shown that bacteriemic infections post-TRUS-B are often caused by *E. coli* exhibiting ciprofloxacin MIC above ECOFF, but below the clinical breakpoint for resistance (i.e., in the range > 0.06–0.5 mg/L). Unpublished data from our group also shows that such low-level ciprofloxacin resistance in *E. coli* colonizing the rectum is an independent risk factor for post-TRUS-B infection, even when a high-dose regimen of ciprofloxacin prophylaxis has been used, adding to the risk posed by fully resistant isolates (MIC > 0.5 mg/L) (personal communication; Jon Forsberg et al., unpublished data). This has recently been corroborated in a study by Kalalahti et al. [15]. Thus, the aim of this study was to determine which quinolone, by disk diffusion, best discriminates phenotypical ciprofloxacin wild-type isolates of *E. coli* (determined by broth microdilution), from isolates with low- and high-level ciprofloxacin resistance, irrespective of the resistance mechanism. The resistance genotype for each isolate was consequently also determined.

## Materials and methods

### Bacterial isolates

#### TRUS-B-related isolates

Isolates were mainly collected from patients undergoing TRUS-B at the Department of Urology, Östergötland County, comprising 31 *E. coli* isolates from blood cultures post-TRUS-B, and 38 *E. coli* isolates obtained from patients immediately before TRUS-B biopsy (eight *E. coli* isolates from urinary samples and 30 nalidixic acid-resistant *E. coli* isolates from fecal samples). Isolates were collected from 2009 to 2014. Only one isolate per patient was included. If several isolates were obtained from one patient, the isolate with the highest ciprofloxacin MIC was included. Species determination was performed with MALDI-TOF mass spectrometry (Brüker, Billerica, MA, USA).

The Regional Ethical Review Board in Linköping, Sweden, approved the study (ref. nos. 2012/2019-31 and 2015/68-32).

#### Additional isolates

In order to enhance the number of isolates with plasmid-mediated quinolone resistance without a concomitant chromosomal resistance determinant, as well as the number of wild-type isolates, isolates from two previous studies were screened for resistance mechanisms (see the “Molecular methods” section). From the study of Karah et al., 35 isolates were screened and one isolate carrying *qnrS* was included [16]. Similarly, 107 ESBL-producing *E. coli* isolates from a study by Östholm Balkhed et al. were screened, and 38 of these were included [17].

#### Control strains

Six control strains were used: *E. coli* ATCC 25922 (wild-type *gyrA* and *parC*), *K. pneumoniae* CCUG 59349 (*qnrB*), *K. pneumoniae* CCUG 59358 (*qnrS1* and *AAC*(*6′*)-*lb*-*cr*), *E. coli* Lo (*qnr A*) from Prof. P. Nordmann, Hôspital Bicêtre, France, *E. coli* DH10B (*qnC*), and *E. coli* TG1 (*qnrD*) both from Dr. L. Cavaco, National Food Institute, Denmark.

### Susceptibility testing

Disk diffusion and ciprofloxacin MIC determination by broth microdilution were used according to EUCAST methodology [14]. The disks used were ciprofloxacin 5 μg, levofloxacin 5 μg, moxifloxacin 5 μg, nalidixic acid 30 μg, and pefloxacin 5 μg (Oxoid, Hampshire, UK). The disks were placed on Mueller Hinton agar plates (Oxoid, Hampshire, UK) inoculated with bacteria (0.5 McFarland) and were incubated for 16–20 h at 36 °C in normal air. Disk diffusion zones and MICs were interpreted according to the EUCAST clinical breakpoints, where available. For pefloxacin, the breakpoint for screening of low-level resistance in *Salmonella* spp. was used (i.e., a 5 μg pefloxacin disk and employing a breakpoint of S ≥ 24 mm) as no breakpoints for other *Enterobacterales* are provided. For nalidixic acid, the epidemiological cutoff value (ECOFF) as defined by EUCAST (> 19 mm) until January 1, 2019, was used (Table 1) [14].

**Table 1:** Sensitivity and specificity of different quinolone disks, when EUCAST breakpoints or ECOFFs are applied, to correctly classify ciprofloxacin non-wild-type isolates as I or R. Non-wild-type MIC defined as ciprofloxacin MIC > 0.064 mg/L in broth microdilution (EUCAST gold standard)

Disk diffusion	Type of breakpoint	Zone diameter (mm)	Sensitivity (%)	Specificity (%)
Ciprofloxacin disk 5 μg	EUCAST clinical breakpoint	S ≥ 25; R < 22	59	100
	EUCAST ECOFF	Wild-type ≥ 25	59	100
Levofloxacin disk 5 μg	EUCAST clinical breakpoint	S ≥ 23; R < 19	46	100
	EUCAST ECOFF	Wild-type ≥ 25	69	100
Moxifloxacin disk 5 μg	EUCAST clinical breakpoint	S ≥ 22; R < 22	59	100
	EUCAST ECOFF	Wild-type ≥ 23	66	100
Nalidixic acid disk 30 μg	EUCAST ECOFF	Wild-type ≥ 19	97	100
Pefloxacin disk 5 μg	Screening breakpoint for quinolone resistance in *Salmonella* spp.	S ≥ 24; R < 24	97	100

For ciprofloxacin MIC determination, the Sensititre® broth microdilution plate DKMGN (Thermo Fisher Scientific, Göteborg, Sweden) was used, according to the manufacturer’s instructions. Ciprofloxacin concentrations were available from 0.06 to 2 mg/L.

Isolated colonies were suspended in NaCl, and turbidity was adjusted to 0.5 McFarland. From the suspension, 10 μL was transferred into 11 mL Müller-Hinton broth and mixed. From the MH broth, 50 μL was transferred to each well. The plate was sealed and incubated at 35 °C for 16–20 h. Following incubation, the plates were visually read. The MIC values were determined as the lowest antibiotic concentration that inhibited microbial growth.

### Molecular methods

Bacterial DNA from the isolates was extracted using Genovision M48 (Qiagen, Hilden, Germany) and the MagAttract DNA Mini M48 kit (Qiagen).

Chromosomal quinolone resistance was identified by specific PCRs using the target sequences: *gyrA* and *parC*. The primers used were described in a previous study [18]. Amplicons were detected by capillary gel electrophoresis using the QIAxcel system and QIAxcel High Resolution Kit (Qiagen). The amplicons were sent to Eurofins MWG Operon (Ebersberg, Germany) for sequencing. The sequences were analyzed to determine the presence of single nucleotide polymorphisms by CLC Main Workbench (Qiagen) and were compared to a reference gene (*gyrA* compared to NP_416734 and *parC* to NP_417491, both from the *E. coli* strain K-12 MG1655) as well as to *gyrA* and *parC* isolated from a control isolate (ATCC 25922).

To identify plasmid-mediated quinolone resistance mechanisms, specific PCRs were aimed at *qnrA*, *qnrB*, *qnrC*, *qnrD*, *qnrS*, and *aac*(*6′*)-*Ib*. The primers used were described in a previous study [19]. The amplicons were detected by capillary gel electrophoresis and were sent for sequencing (Eurofins MWG Operon) and confirmed by matching to reference genes found in GenBank (National Center for Biotechnology Information): *qnrA1* AY070235, *qnrB1* DQ351241, *qnrC* EU917444, *qnrD* FJ228229, *qnrS1* AB187515, and *aac*-(*6′*)-*Ib* L25666. The modified gene of aminoglycoside-modifying enzyme *aac*(*6′*)-*Ib*-*cr* contains two amino acid substitutions as compared to wild-type *aac*(*6′*)-*Ib*. To find these substitutions, all the *aac*(*6′*)-*Ib* positive sequences were analyzed by CLC Main Workbench and compared to a control strain CCUG59358.

Primers (Supplemental Table 1) were ordered from Eurofins MWG Operon. All forward primers were tagged with M13 uni-21 tags and reverse primers for *gyrA* and *parC* were tagged with SP6 tags. All PCR used the HotStarTaq Master Mix (Qiagen) in a final reaction volume of 25 μL. The PCR reaction was initiated with 15 min of denaturation at 95 °C. This was followed by 30 cycles consisting of 30 s of denaturation at 95 °C, 20 s of annealing at 55 °C (58 °C for *qnr C* and *qnr D*), and 30 s of extension at 72 °C. The final step was 8 min of extension at 72 °C. Positive and negative controls were used in every PCR run. The positive controls were the control strains mentioned previously. The negative controls were phosphate-buffered saline having undergone the DNA extraction reaction.

### Sensitivity and specificity

The ability of the five quinolones using the disk diffusion and EUCAST breakpoints, to discriminate wild-type isolates from non-wild-type isolates was calculated in a binary classification test (2 × 2 contingency table) and expressed as sensitivity and specificity. That is, sensitivity describes the probability that a certain disk diffusion test, using EUCAST breakpoints, will classify an isolate as non-susceptible (I + R (+ATU, when applicable)) when ciprofloxacin MIC is > 0.06 mg/L (i.e., phenotypically non-wild-type). Specificity describes the probability that the disk diffusion test, using EUCAST breakpoints, will classify an isolate as susceptible (S) when ciprofloxacin MIC is ≤ 0.06 mg/L (i.e., phenotypically wild-type).

## Results

### Screening with disk diffusion

The distributions of zone diameters related to ciprofloxacin MIC are shown in Supplemental Table 2. The present zone diameter clinical breakpoint for ciprofloxacin 5 μg (S ≥ 25 mm), which coincides with the ciprofloxacin disk ECOFF (25 mm) resulted in 59% sensitivity, and a specificity of 100% (Table 1 and Supplemental Table 2). Using a levofloxacin 5 μg disk (S ≥ 23 mm) or moxifloxacin disk 5 μg (S ≥ 22 mm) and applying clinical breakpoints, yielded sensitivities of 46% and 59%, respectively and a 100% specificity for both substances (Table 1 and Supplemental Table 2). Applying ECOFFs (levofloxacin 25 mm, moxifloxacin 23 mm) resulted in slightly better sensitivities, 69% and 66%, respectively, leaving specificity unchanged.

The nalidixic acid 30 μg EUCAST ECOFF (S ≥ 19 mm) resulted in a sensitivity of 97% and specificity of 100%. Using a 5 μg pefloxacin disk and employing a breakpoint of S ≥ 24 mm resulted in a 97% sensitivity and 100% specificity in the present set of *E. coli* isolates (Table 1 and Supplemental Table 2).

### Quinolone resistance genotype

Out of the 108 isolates screened, no resistance mechanisms were detected in 40 isolates. Thirty were first-step mutants (*gyrA*) without plasmid-mediated resistance. Twenty-two were double mutants (*gyrA* and *parC*) without plasmid-mediated resistance. One of these double mutants also produced the *AAC* amplicon but *AAC*(*6′*)-*lb*-*cr* could not be verified by DNA sequencing (presented just as *gyrA* + *parC* in tables).

Three, ten, and one isolates carried the *qnrB* gene, the *qnrS* gene, and the *AAC*(*6′*)-*lb*-*cr* gene, respectively, all without mutations in the target genes (*gyrA*/*parC*). One first-step mutant also carried the *AAC*(*6′*)-*lb*-*cr* gene. One isolate was a double mutant carrying the *AAC*(*6′*)-*lb*-*cr* gene (Table 2 and Supplemental Table 3).

**Table 2:** Ciprofloxacin MIC of 108 *E. coli* isolates and their corresponding genotypes

MIC	No detected resistance gene	*gyrA*	*gyrA+ parC*	*qnrB1*	*Qnr S1*	*aac-lb*	*gyrA+ AAC*	*gyrApParC+ aac*
≤ 0.06	38				1	1		
0.12		3						
0.25	1	24		1	3			
0.5		2		2	3		1	
1					1			
2					1			
> 2			23		1			1

All isolates where no resistance mechanism was detected, except one, had a ciprofloxacin MIC of ≤ 0.06 mg/L. As expected, all first-step mutants had MICs in the range 0.12–0.5 mg/L and all isolates with mutations in both *gyrA* and *parC* had MICs of > 2 mg/L. The majority, 9 of 14 isolates with single plasmid-mediated resistance mechanisms *(qnrB*, *qnrS*, or *AAC*(*6′*)-*lb*-*cr*) showed MICs in the low-level range (0.12–0.5 mg/L). However, two isolates (1 *qnrB*, 1 *qnrS*) had ciprofloxacin MIC of ≤ 0.06 mg/L. These two isolates were interpreted as susceptible by disk diffusion no matter which quinolone was used for screening. Three isolates (all *qnrS*) had MICs in the resistant range (> 0.5 mg/L) (Table 2 and Supplemental Table 3).

The distributions of disk diffusion zone diameters related to genotype are presented in Supplemental Table 3.

## Discussion

During recent years, many studies have shown the importance of rectal colonization with ciprofloxacin-resistant *E. coli* as a risk factor for post-TRUS-B infection [11, 12]. However, definitions of ciprofloxacin resistance and microbiological methods vary among these reports. In a study by Kalalahti et al., in a setting where ciprofloxacin prophylaxis (one single dose of 750 mg) was used, among the seven men with infections that were confirmed by culture and caused by *E. coli*, pre-biopsy rectal cultures grew *E. coli* in the low-level resistance range (0.094–0.5 mg/L) in four cases (i.e., non-wild-type); the remaining three men were colonized by *E. coli* with MICs of > 32 mg/L. *E. coli* isolated from urine and blood during the subsequent infections had corresponding susceptibility profiles [15]. In order to find the best way to screen for such *E. coli* in fecal samples obtained pre-biopsy, we aimed to identify the quinolone that best discriminates wild-type isolates of *E. coli* from isolates with low- and high-level resistance, irrespective of resistance mechanisms.

All quinolone disks readily identified isolates with a wild-type phenotype at a specificity of 100%. However, sensitivity, i.e., the ability to identify an isolate with a non-wild-type ciprofloxacin MIC as non-susceptible, varied substantially from 46% (levofloxacin clinical breakpoint) to 97% (nalidixic acid ECOFF and pefloxacin). Using the nalidixic acid ECOFF, all isolates with mutations in *gyrA*, with or without concomitant mutations in *parC*, were identified as non-wild-type. This has been shown earlier [20, 21]. However, 4 of 14 isolates with plasmid-mediated quinolone resistance (PMQR) mechanisms were identified as phenotypic wild-type using the nalidixid acid ECOFF. Two of these had ciprofloxacin MIC corresponding to a wild-type phenotype.

There are no EUCAST breakpoints regarding *E. coli* and pefloxacin susceptibility. However, applying 5 μg pefloxacin disk and employing a breakpoint of S ≥ 24 mm, only two isolates with non-wild-type ciprofloxacin MIC were incorrectly identified as susceptible. These two isolates were both first-step mutants. In addition, two isolates with PMQR (1 *qnrB*, 1 *qnrS*) with ciprofloxacin MICs of ≤ 0.06 mg/L were identified as susceptible by pefloxacin disk screening. Combining the results from both the nalidixic disk and the pefloxacin screening, with a result of non-susceptibility from either of the two interpreted as a non-susceptible isolate, would render 100% sensitivity and 100% specificity (Supplemental Table 3).

The sensitivity of the ciprofloxacin disk, applying the current clinical breakpoint for susceptibility, was only 59%. However, this is not surprising as this breakpoint is designed to determine isolates with an MIC of ≤ 0.25 mg/L. Fifty-eight out of 61 isolates that were considered susceptible when using the ciprofloxacin disk had ciprofloxacin MICs of ≤ 0.25 mg/L, i.e., results from disk diffusion were concurrent with the broth dilution method. The remaining three isolates all had MICs of 0.5 mg/L (Supplemental Table 2). However, using the ECOFF millimeter zone for ciprofloxacin when screening of isolates with non-wild-type MICs did not improve the sensitivity as the disk diffusion ECOFF currently is the same as the breakpoint for susceptibility (≥ 25 mm). Almost all first-step mutants, and 6 of 15 PMQR carriers, were classified as susceptible with the current ciprofloxacin MIC breakpoint (Table 2). The clinical importance of PMQR conveying low-level resistance remains to be explored in clinical studies. However, in experimental models of pneumonia and urinary tract infection, the bactericidal effect of ciprofloxacin was greatly reduced and mortality increased in the presence of *qnr* and AAC(6′)-lb-cr elements [22–24].

Quinolone resistance in *E. coli* is most commonly caused by point mutations in the genes coding for the target enzymes, DNA gyrase and topoisomerase IV. The present study corroborates this as only 7 of 69 TRUS-B-related isolates carried any PMQR determinant (data not shown). In order to fulfill the aim of the study, additional isolates had to be included. The great heterogeneity of the material offered a large variety of resistance mechanisms and a wide distribution of susceptibility. Although there is no scarcity of studies of men with subsequent infections after TRUS-B, the frequency of different quinolone resistance determinants in *E. coli* causing these infections has rarely been studied. In studies on isolates of *E. coli* in other populations, the frequency of plasmid-mediated quinolone resistance determinants varies between 1 and 15% in different populations [16, 25, 26]. In studies where a higher number of ESBL-producing isolates are included, the frequency of plasmid-mediated quinolone resistance is generally higher [27].

A first mutation in the gene *gyrA* alters the binding site of DNA gyrase, the primary target of quinolones in *E. coli*, and confers low to moderate levels of resistance (ciprofloxacin MIC 0.125–1 mg/L) [28]. Additional mutations in *gyrA* and also mutations in a second gene, *parC*, coding for the binding site of topoisomerase IV, have been shown to then cause high-level resistance [29] . Whereas mutations in *parC*, when added to mutations in *gyrA*, inevitably caused high-level resistance, secondary mutations in *gyrA* were only found in two isolates in the present study, and these did not affect ciprofloxacin MIC (Supplemental Table 3). Levels of susceptibility depending on the specific site of a single nucleotide polymorphism (SNP) have also been shown, but again, not in this collection [30]. The site of the first-step mutation was almost exclusively the S83L polymorphism (Supplemental Table 1).

## Conclusion

The nalidixic acid disk and the pefloxacin disk were both successful in screening for *E. coli* phenotypically ciprofloxacin wild-type. We suggest that in situations where low-level quinolone resistance might be of importance, such as when screening for quinolone resistance in fecal samples pre-TRUS-B, a pefloxacin (S ≥ 24 mm) or nalidixid acid (S ≥ 19 mm) disk, or a combination of the two, should be used. In a setting where PMQR is prevalent, pefloxacin might perform better than nalidixid acid.

## Electronic supplementary material

ESM 1 (DOCX 16 kb)

ESM 2 (DOCX 26 kb)

ESM 3 (XLSX 16 kb)

## References

[1] Lindstedt S, Lindstrom U, Ljunggren E, Wullt B, Grabe M (2006). Single-dose antibiotic prophylaxis in core prostate biopsy: impact of timing and identification of risk factors. Eur Urol.

[2] Otrock ZK, Oghlakian GO, Salamoun MM, Haddad M, Bizri AR (2004). Incidence of urinary tract infection following transrectal ultrasound guided prostate biopsy at a tertiary-care medical center in Lebanon. Infect Control Hosp Epidemiol.

[3] Zaytoun OM, Vargo EH, Rajan R, Berglund R, Gordon S, Jones JS (2011). Emergence of fluoroquinolone-resistant Escherichia coli as cause of postprostate biopsy infection: implications for prophylaxis and treatment. Urology.

[4] Kapoor DA, Klimberg IW, Malek GH, Wegenke JD, Cox CE, Patterson AL, Graham E, Echols RM, Whalen E, Kowalsky SF (1998). Single-dose oral ciprofloxacin versus placebo for prophylaxis during transrectal prostate biopsy. Urology.

[5] Carignan A, Roussy JF, Lapointe V, Valiquette L, Sabbagh R, Pepin J (2012). Increasing risk of infectious complications after transrectal ultrasound-guided prostate biopsies: time to reassess antimicrobial prophylaxis?. Eur Urol.

[6] Nam RK, Saskin R, Lee Y, Liu Y, Law C, Klotz LH, Loblaw DA, Trachtenberg J, Stanimirovic A, Simor AE, Seth A, Urbach DR, Narod SA (2013). Increasing hospital admission rates for urological complications after transrectal ultrasound guided prostate biopsy. J Urol.

[7] Lundstrom KJ, Drevin L, Carlsson S, Garmo H, Loeb S, Stattin P, Bill-Axelson A (2014). Nationwide population based study of infections after transrectal ultrasound guided prostate biopsy. J Urol.

[8] Feliciano J, Teper E, Ferrandino M, Macchia RJ, Blank W, Grunberger I, Colon I (2008). The incidence of fluoroquinolone resistant infections after prostate biopsy--are fluoroquinolones still effective prophylaxis?. J Urol.

[9] Rudzinski JK, Kawakami J (2014). Incidence of infectious complications following transrectal ultrasound-guided prostate biopsy in Calgary, Alberta, Canada: a retrospective population-based analysis. Can Urol Assoc J.

[10] Williamson DA, Barrett LK, Rogers BA, Freeman JT, Hadway P, Paterson DL (2013). Infectious complications following transrectal ultrasound-guided prostate biopsy: new challenges in the era of multidrug-resistant Escherichia coli. Clin Infect Dis.

[11] Scott S, Harris PN, Williamson DA, Liss MA, Doi SAR, Roberts MJ (2018). The effectiveness of targeted relative to empiric prophylaxis on infectious complications after transrectal ultrasound-guided prostate biopsy: a meta-analysis. World J Urol.

[12] Steensels D, Slabbaert K, De Wever L, Vermeersch P, Van Poppel H, Verhaegen J (2012). Fluoroquinolone-resistant E. coli in intestinal flora of patients undergoing transrectal ultrasound-guided prostate biopsy--should we reassess our practices for antibiotic prophylaxis?. Clin Microbiol Infect.

[13] Lee K, Drekonja DM, Enns EA (2018). Cost-effectiveness of antibiotic prophylaxis strategies for transrectal prostate biopsy in an era of increasing antimicrobial resistance. Value Health.

[14] EUCAST (2019). Clinical breakpoints. http://www.eucast.org/clinical_breakpoints/. Last accessed 2019-01-25

[15] Kalalahti I, Huotari K, Lahdensuo K, Tarkka E, Santti H, Rannikko A, Patari-Sampo A (2018). Rectal E. coli above ciprofloxacin ECOFF associate with infectious complications following prostate biopsy. Eur J Clin Microbiol Infect Dis.

[16] Karah N, Poirel L, Bengtsson S, Sundqvist M, Kahlmeter G, Nordmann P, Sundsfjord A, Samuelsen O, Norwegian Study Group on P (2010). Plasmid-mediated quinolone resistance determinants qnr and aac(6′)-Ib-cr in Escherichia coli and Klebsiella spp. from Norway and Sweden. Diagn Microbiol Infect Dis.

[17] Ostholm-Balkhed A, Tarnberg M, Nilsson M, Nilsson LE, Hanberger H, Hallgren A, Travel Study Group of Southeast S (2013). Travel-associated faecal colonization with ESBL-producing Enterobacteriaceae: incidence and risk factors. J Antimicrob Chemother.

[18] Nawaz M, Sung K, Kweon O, Khan S, Nawaz S, Steele R (2015). Characterisation of novel mutations involved in quinolone resistance in Escherichia coli isolated from imported shrimp. Int J Antimicrob Agents.

[19] Ostholm Balkhed A, Tarnberg M, Monstein HJ, Hallgren A, Hanberger H, Nilsson LE (2013). High frequency of co-resistance in CTX-M-producing Escherichia coli to non-beta-lactam antibiotics, with the exceptions of amikacin, nitrofurantoin, colistin, tigecycline, and fosfomycin, in a county of Sweden. Scand J Infect Dis.

[20] Cavaco LM, Aarestrup FM (2009). Evaluation of quinolones for use in detection of determinants of acquired quinolone resistance, including the new transmissible resistance mechanisms qnrA, qnrB, qnrS, and aac(6')Ib-cr, in Escherichia coli and Salmonella enterica and determinations of wild-type distributions. J Clin Microbiol.

[21] Skov R, Matuschek E, Sjolund-Karlsson M, Ahman J, Petersen A, Stegger M, Torpdahl M, Kahlmeter G (2015). Development of a pefloxacin disk diffusion method for detection of fluoroquinolone-resistant Salmonella enterica. J Clin Microbiol.

[22] Allou N, Cambau E, Massias L, Chau F, Fantin B (2009). Impact of low-level resistance to fluoroquinolones due to qnrA1 and qnrS1 genes or a gyrA mutation on ciprofloxacin bactericidal activity in a murine model of Escherichia coli urinary tract infection. Antimicrob Agents Chemother.

[23] Dominguez-Herrera J, Velasco C, Docobo-Perez F, Rodriguez-Martinez JM, Lopez-Rojas R, Briales A, Pichardo C, Diaz-de-Alba P, Rodriguez-Bano J, Pascual A, Pachon J (2013). Impact of qnrA1, qnrB1 and qnrS1 on the efficacy of ciprofloxacin and levofloxacin in an experimental pneumonia model caused by Escherichia coli with or without the GyrA mutation Ser83Leu. J Antimicrob Chemother.

[24] Guillard T, Cambau E, Chau F, Massias L, de Champs C, Fantin B (2013). Ciprofloxacin treatment failure in a murine model of pyelonephritis due to an AAC(6′)-Ib-cr-producing Escherichia coli strain susceptible to ciprofloxacin in vitro. Antimicrob Agents Chemother.

[25] Jeong HS, Bae IK, Shin JH, Kim SH, Chang CL, Jeong J, Kim S, Lee CH, Ryoo NH, Lee JN (2011). Fecal colonization of Enterobacteriaceae carrying plasmid-mediated quinolone resistance determinants in Korea. Microb Drug Resist.

[26] Kim HB, Park CH, Kim CJ, Kim EC, Jacoby GA, Hooper DC (2009). Prevalence of plasmid-mediated quinolone resistance determinants over a 9-year period. Antimicrob Agents Chemother.

[27] Rodriguez-Martinez JM, Machuca J, Cano ME, Calvo J, Martinez-Martinez L, Pascual A (2016). Plasmid-mediated quinolone resistance: two decades on. Drug Resist Updat.

[28] Hoshino K, Kitamura A, Morrissey I, Sato K, Kato J, Ikeda H (1994). Comparison of inhibition of Escherichia coli topoisomerase IV by quinolones with DNA gyrase inhibition. Antimicrob Agents Chemother.

[29] Jacoby GA (2005). Mechanisms of resistance to quinolones. Clin Infect Dis.

[30] Fu Y, Zhang W, Wang H, Zhao S, Chen Y, Meng F, Zhang Y, Xu H, Chen X, Zhang F (2013). Specific patterns of gyrA mutations determine the resistance difference to ciprofloxacin and levofloxacin in Klebsiella pneumoniae and Escherichia coli. BMC Infect Dis.

