# Supplementary material for: Phenotypic screening for quinolone resistance in Escherichia coli
Source: Eur J Clin Microbiol Infect Dis. 2019 Jun 18;38(9):1765–71. doi: 10.1007/s10096-019-03608-w (PMC6695352; doi:10.1007/s10096-019-03608-w)
Supplement: Supplementary file 1 — (DOCX 16 kb) [file 10096_2019_3608_MOESM1_ESM.docx]

Table S1. Primers used in this study (bold). ^a^M13 uni-21 sequence (roman) for sequencing used in all forward primers. ^b^Sp6 sequence (roman) for sequencing used in *gyrA* and parC reverse primers.

| **Primer** | **Nucleotide sequence 5´- 3´** | **Target gene** | **Size (bp)** |
| --- | --- | --- | --- |
| *gyrA* F-M13 | TGT AAA ACG ACG GCC AGT^a^ **AAA TCT GCC CGT GTC GTT GGT** | *gyrA* | 344 |
| *gyrA* R-SP6 | C ATT TAG GTG ACA CTA TAG^b^ **GCC ATA CCT ACG GCG ATA CC** |  |  |
| *parC* F-M13 | TGT AAA ACG ACG GCC AGT^a^ **CTG AAT GCC AGC GCC AAA TT** | *parC* | 168 |
| *parC* R-SP6 | C ATT TAG GTG ACA CTA TAG^b^ **GCG AAC GAT TTC GGA TCG TC** |  |  |
| *qnrA* F-M13 | TGT AAA ACG ACG GCC AGT^a^ **TCA GCA AGA GGA TTT CTC ACG CCA** | *qnrA* | 549 |
| *qnrA* R | **TCC AGA TCG GCA AAG GTA AGG TCA** |  |  |
| *qnrB* F-M13 | TGT AAA ACG ACG GCC AGT^a^ **GG Y ACT GAA TTT AT Y GGC TG Y C** | *qnrB* | 260 |
| *qnrB* R | **GTG ATA TA K GC R CT R CAA AAC CA** |  |  |
| *qnrC* F-M13 | TGT AAA ACG ACG GCC AGT^a^ **ATG CAG ACC TAC GAG ATG CTT C** | *qnrC* | 353 |
| *qnrC* R | **GCA TTG CTC CCA AAA GTC ATC AG** |  |  |
| *qnrD* F-M13 | TGT AAA ACG ACG GCC AGT^a^ **CAG GAA TAG CTT GGA AGG GTG TG** | *qnrD* | 297 |
| *qnrD* R | **CGA TTT TCC CAC AGT TCG CAC** |  |  |
| *qnrS* F-M13 | TGT AAA ACG ACG GCC AGT^a^ **GGA AAC CTA C MR TCA TAC ATA TCG** | *qnrS* | 489 |
| *qnrS* R | **TCT GAC TCT TTC AGT GAT GC** |  |  |
| *aac* F-M13 | TGT AAA ACG ACG GCC AGT^a^ **GAC ACT TGC TGA CGT ACA GGA ACA G** | *aac(6’)-lb* | 416 |
| *aac* R | **GTG TTT GAA CCA TGT ACA CGG CTG G** |  |  |
