# Supplementary material for: Phenotypic screening for quinolone resistance in Escherichia coli
Source: Eur J Clin Microbiol Infect Dis. 2019 Jun 18;38(9):1765–71. doi: 10.1007/s10096-019-03608-w (PMC6695352; doi:10.1007/s10096-019-03608-w)
Supplement: Supplementary file 2 — (DOCX 26 kb) [file 10096_2019_3608_MOESM2_ESM.docx]

| Ciprofloxacin  **Zone (mm)** | | **6** | **7** | **8** | **9** | **10** | **11** | **12** | **13** | **14** | **15** | **16** | **17** | **18** | **19** | **20** | **21** | **22** | **23** | **24** | **25** | **26** | **27** | **28** | **29** | **30** | **31** | **32** | **33** | **34** | **35** |
| --- | --- | --- | --- | --- | --- | --- | --- | --- | --- | --- | --- | --- | --- | --- | --- | --- | --- | --- | --- | --- | --- | --- | --- | --- | --- | --- | --- | --- | --- | --- | --- |
| Ciprofloxacin MIC (mg/L) | ≤ 0.06 |  |  |  |  |  |  |  |  |  |  |  |  |  |  |  |  |  |  |  |  |  |  | 2 |  | 6 | 1 | 13 | 3 | 9 | 6 |
|  | 0.12 |  |  |  |  |  |  |  |  |  |  |  |  |  |  |  |  |  |  |  |  |  | 2 |  | 1 |  |  |  | 1 |  |  |
|  | 0.25 |  |  |  |  |  |  | 1 |  |  | 1 |  |  |  |  |  |  |  | 2 | 7 | 4 | 4 | 5 | 3 | 2 |  |  |  |  |  |  |
|  | 0.5 |  |  |  |  |  |  |  |  |  |  |  |  |  |  |  |  | 2 |  | 1 | 2 | 1 | 1 |  |  | 1 |  |  |  |  |  |
|  | 1 |  |  |  |  |  |  |  |  |  |  |  |  |  |  |  |  |  |  |  | 1 |  |  |  |  |  |  |  |  |  |  |
|  | ≥ 2 | 18 | 2 | 1 |  | 2 |  |  | 1 | 1 |  |  | 1 |  |  |  |  |  |  |  |  |  |  |  |  |  |  |  |  |  |  |
| Levofloxacin  **Zone (mm)** | | **6** | **7** | **8** | **9** | **10** | **11** | **12** | **13** | **14** | **15** | **16** | **17** | **18** | **19** | **20** | **21** | **22** | **23** | **24** | **25** | **26** | **27** | **28** | **29** | **30** | **31** | **32** | **33** | **34** | **35** |
| Ciprofloxacin MIC (mg/L) | ≤ 0.06 |  |  |  |  |  |  |  |  |  |  |  |  |  |  |  |  |  |  |  |  |  | 1 | 7 | 3 | 5 | 9 | 5 | 8 | 2 |  |
|  | 0.12 |  |  |  |  |  |  |  |  |  |  |  |  |  |  |  |  |  |  |  | 2 |  | 1 |  |  |  |  |  | 1 |  |  |
|  | 0.25 |  |  |  |  |  |  |  |  | 1 |  |  |  |  |  |  |  | 2 | 7 | 7 | 1 | 6 | 5 |  |  |  |  |  |  |  |  |
|  | 0.5 |  |  |  |  |  |  |  |  |  |  |  |  |  |  |  | 1 | 1 | 1 | 1 | 1 | 2 |  |  | 1 |  |  |  |  |  |  |
|  | 1 |  |  |  |  |  |  |  |  |  |  |  |  |  |  |  |  |  |  |  | 1 |  |  |  |  |  |  |  |  |  |  |
|  | ≥ 2 | 10 |  | 1 | 3 | 6 | 3 |  |  | 1 |  | 1 | 1 |  |  |  |  |  |  |  |  |  |  |  |  |  |  |  |  |  |  |
| Moxifloxacin  **Zone (mm)** | | **6** | **7** | **8** | **9** | **10** | **11** | **12** | **13** | **14** | **15** | **16** | **17** | **18** | **19** | **20** | **21** | **22** | **23** | **24** | **25** | **26** | **27** | **28** | **29** | **30** | **31** | **32** | **33** | **34** | **35** |
| Ciprofloxacin MIC (mg/L) | ≤ 0.06 |  |  |  |  |  |  |  |  |  |  |  |  |  |  |  |  |  |  |  | 2 | 5 | 7 | 5 | 4 | 9 |  | 6 | 1 | 1 |  |
|  | 0.12 |  |  |  |  |  |  |  |  |  |  |  |  |  |  |  | 1 |  | 2 |  |  |  |  |  | 1 |  |  |  |  |  |  |
|  | 0.25 |  |  |  |  |  |  |  |  |  |  |  |  | 1 | 2 |  | 4 | 5 | 6 | 5 | 3 | 2 | 1 |  |  |  |  |  |  |  |  |
|  | 0.5 |  |  |  |  | 1 |  |  |  |  |  | 1 |  | 1 | 1 | 1 |  |  | 3 |  |  |  |  |  |  |  |  |  |  |  |  |
|  | 1 |  |  |  |  |  |  |  |  |  |  |  |  |  |  | 1 |  |  |  |  |  |  |  |  |  |  |  |  |  |  |  |
|  | ≥ 2 | 12 |  | 6 | 4 | 1 | 1 |  | 1 |  | 1 |  |  |  |  |  |  |  |  |  |  |  |  |  |  |  |  |  |  |  |  |
| Nalidixic acid  **Zone (mm)** | | **6** | **7** | **8** | **9** | **10** | **11** | **12** | **13** | **14** | **15** | **16** | **17** | **18** | **19** | **20** | **21** | **22** | **23** | **24** | **25** | **26** | **27** | **28** | **29** | **30** | **31** | **32** | **33** | **34** | **35** |
| Ciprofloxacin MIC (mg/L) | ≤ 0.06 |  |  |  |  |  |  |  |  |  |  |  |  |  |  |  |  | 3 | 9 | 8 | 9 | 3 | 6 | 2 |  |  |  |  |  |  |  |
|  | 0.12 | 3 | 1 |  |  |  |  |  |  |  |  |  |  |  |  |  |  |  |  |  |  |  |  |  |  |  |  |  |  |  |  |
|  | 0.25 | 22 | 1 | 1 |  |  | 1 |  |  |  | 2 |  |  |  |  | 2 |  |  |  |  |  |  |  |  |  |  |  |  |  |  |  |
|  | 0.5 | 3 |  |  |  |  |  | 1 |  | 2 |  | 2 |  |  |  |  |  |  |  |  |  |  |  |  |  |  |  |  |  |  |  |
|  | 1 |  |  |  | 1 |  |  |  |  |  |  |  |  |  |  |  |  |  |  |  |  |  |  |  |  |  |  |  |  |  |  |
|  | ≥ 2 | 26 |  |  |  |  |  |  |  |  |  |  |  |  |  |  |  |  |  |  |  |  |  |  |  |  |  |  |  |  |  |
| Pefloxacin  **Zone (mm)** | | **6** | **7** | **8** | **9** | **10** | **11** | **12** | **13** | **14** | **15** | **16** | **17** | **18** | **19** | **20** | **21** | **22** | **23** | **24** | **25** | **26** | **27** | **28** | **29** | **30** | **31** | **32** | **33** | **34** | **35** |
| Ciprofloxacin MIC (mg/L) | ≤ 0.06 |  |  |  |  |  |  |  |  |  |  |  |  |  |  |  |  |  |  |  | 1 | 4 | 2 | 10 | 9 | 10 | 2 | 2 |  |  |  |
|  | 0.12 |  |  |  |  |  |  |  |  |  |  |  |  |  |  | 1 |  | 2 |  |  | 1 |  |  |  |  |  |  |  |  |  |  |
|  | 0.25 | 1 |  |  |  |  |  |  |  |  |  |  | 1 | 3 | 6 | 8 | 5 | 2 | 2 |  |  | 1 |  |  |  |  |  |  |  |  |  |
|  | 0.5 |  | 1 |  |  |  |  |  |  |  |  | 2 | 1 |  | 3 |  | 1 |  |  |  |  |  |  |  |  |  |  |  |  |  |  |
|  | 1 |  |  |  |  |  |  |  | 1 |  |  |  |  |  |  |  |  |  |  |  |  |  |  |  |  |  |  |  |  |  |  |
|  | ≥ 2 | 25 |  |  |  |  | 1 |  |  |  |  |  |  |  |  |  |  |  |  |  |  |  |  |  |  |  |  |  |  |  |  |

Supplemental table 2. Ciprofloxacin resistance phenotype (Broth microdilution MIC) in 108 *E. coli* isolates and corresponding inhibition zones of ciprofloxacin, levofloxacin, moxifloxacin, nalidixic acid and pefloxacin using disc diffusion tests.
